# Skin Immunity to Dermatophytes: From Experimental Infection Models to Human Disease

**DOI:** 10.3389/fimmu.2020.605644

**Published:** 2020-12-02

**Authors:** Verónica L. Burstein, Ignacio Beccacece, Lorena Guasconi, Cristian J. Mena, Laura Cervi, Laura S. Chiapello

**Affiliations:** ^1^Laboratorio de Parasitología y Micología Experimental. Departamento de Bioquímica Clínica, Facultad de Ciencias Químicas, Universidad Nacional de Córdoba, Córdoba, Argentina; ^2^Centro de Investigaciones en Bioquímica Clínica e Inmunología (CIBICI), Consejo Nacional de Investigaciones Científicas y Técnicas (CONICET), Córdoba, Argentina

**Keywords:** skin immunity, mycoses, antifungal immunity, fungal pathogenesis, interleukin-17

## Abstract

Dermatophytoses (ringworms) are among the most frequent skin infections and are a highly prevalent cause of human disease worldwide. Despite the incidence of these superficial mycoses in healthy people and the compelling evidence on chronic and deep infections in immunocompromised individuals, the mechanisms controlling dermatophyte invasion in the skin are scarcely known. In the last years, the association between certain primary immunodeficiencies and the susceptibility to severe dermatophytosis as well as the evidence provided by novel experimental models mimicking human disease have significantly contributed to deciphering the basic immunological mechanisms against dermatophytes. In this review, we outline the current knowledge on fungal virulence factors involved in the pathogenesis of dermatophytoses and recent evidence from human infections and experimental models that shed light on the cells and molecules involved in the antifungal cutaneous immune response. The latest highlights emphasize the contribution of C-type lectin receptors signaling and the cellular immune response mediated by IL-17 and IFN-γ in the anti-dermatophytic defense and skin inflammation control.

## Introduction

**Figure 1:**
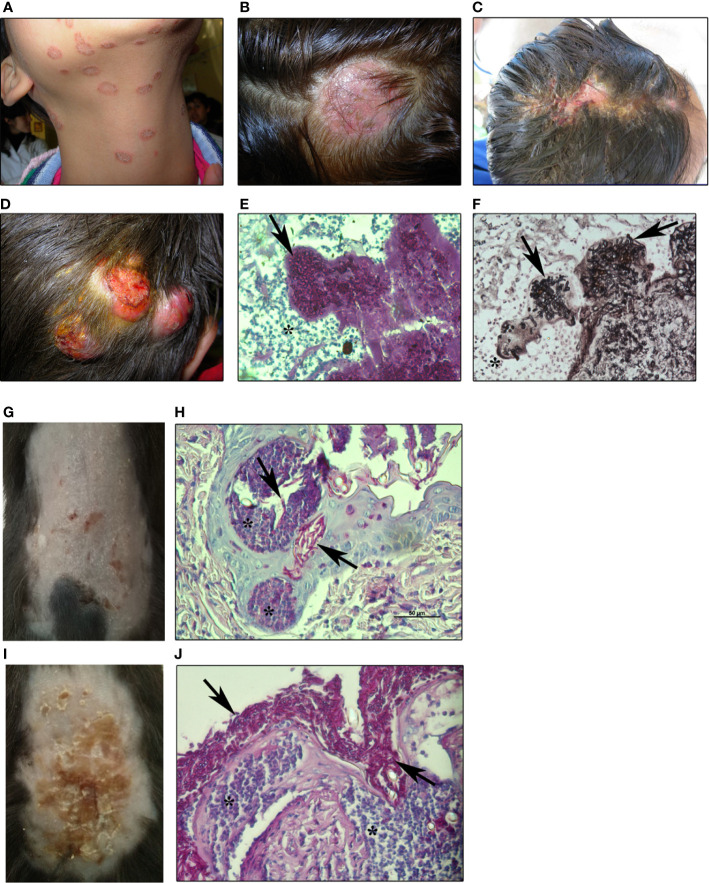
Human and experimental dermatophytosis **(A, B)**. Superficial mild inflammatory infections in humans by *M. canis* **(A)** *tinea corporis* and **(B)** *tinea capitis* by *M. canis* **(C–F)**. Inflammatory human dermatophytosis **(C)**: inflammatory *tinea capitis* (*Kerion de Celsi*) by *T. mentagrophytes* and **(D)** deep infection of the scalp by *M. canis* (ref. 106) showing hyphae in dermis **(E)** stained with PAS-hematoxylin or **(F)** Grocott-Gomori’s methenamine silver (GMS) staining (200x magnification) **(G–J)**. Experimental dermatophytosis after epicutaneous infection of **(G, H)** wild-type (WT) or **(I, J)** IL-17RA-deficient C57BL/6 mice with *M. canis* (8 days post-infection) (ref. 51) **(G)**. Mild inflammatory lesions and **(H)** histopathology showing PAS-positive hyphae invading the *stratum corneum* and hair follicles in WT **(I)**. Highly inflammatory lesions and **(J)** histopathology showing extensive superficial fungal proliferation in IL-17RA-deficient mice. Arrows: *M. canis* hyphae. Asterix: inflammatory reaction (400x magnification). All images are property of Chiapello’s lab.

The skin is the most extensive organ of the body, is an ecological niche for microbiota and the first barrier against aggression from environmental noxa and pathogenic microorganisms. Not only is a physical barrier but also a dynamic system constituted by the skin resident immune system that is crucial to control an infection, resolve damage, or maintain tissue homeostasis. Among the most frequent human skin infections, dermatophytoses (ringworms) represent the fourth cause of disease with a global incidence estimated in 20 to 25% within the healthy population (1–3). These infections are caused by filamentous fungi, ancestrally digesters of soil keratin, that have adapted to the keratinized tissue of mammals, and became parasitic microorganisms to animals and humans. Therefore, dermatophytoses are characterized by hyphae superficial invasion into the skin, hair, and nails causing subacute or chronic infections with different inflammation degrees among immunocompetent individuals (**Figure 1**). Recent taxonomic changes classify dermatophytes into five genera*: Epidermophyton, Trichophyton, Microsporum, Arthroderma*, and *Nannizzia* (4) and, among them, there are different species adapted to particular ecological niches and hosts which led to the classification in geophilic, zoophilic and anthropophilic fungi. Anthropophilic species (*Trichophyton rubrum, Epidermophyton floccosum*) are well adapted to humans and often cause chronic infections with mild clinical symptoms. In contrast, dermatophytes from animals (*Microsporum canis, Trichophyton/Arthroderma benhamiae, Trichophyton mentagrophytes*, etc.) or soil (*Nannizzia gypsea/Microsporum gypseum*) are frequently isolated from patients suffering from mild to highly inflammatory dermatophytosis but with lesions that are prone to spontaneous resolution (5) (**Figure 1**). In contrast, the immunosuppressed population (especially cell-mediated immunity deficiency settings such as HIV-AIDS, transplant, neoplasia, diabetes, or corticosteroid therapy) is particularly susceptible to these infections showing extensive superficial lesions that are often unresponsive to conventional antifungal treatment (3, 6, 7). This was recently observed in India where there was a significant increase in treatment-recalcitrant, recurrent and chronic dermatophytosis probably due to indiscriminate use of antibiotics and corticosteroid drug combination (8).

Despite the high incidence of dermatophytosis in healthy people as well as the complications of these infections in immunocompromised individuals, the immune mechanisms that control dermatophyte invasion are less known and studied than those involved in other fungal diseases. In the last years, the identification of genetic mutations in patients with primary immunodeficiencies associated with severe deep or widespread dermatophytosis and the development of experimental models that mimic human infection have contributed to deciphering the basic mechanisms of cutaneous immunity against dermatophytes. Taking into account the recent evidence from human infections and experimental models, in this review we discuss the latest advances in the knowledge and state-of-the-art of innate and adaptive mechanisms of the immune response against dermatophyte fungi.

## Dermatophyte Virulence Factors

In order to degrade keratin, dermatophytes secrete an arsenal of hydrolytic enzymes (proteases) which are assumed to be the main virulence factors in live tissue infection (9). It is known that the pathogenesis of dermatophytosis includes several stages (i.e. fungal adhesion, germination, invasion, penetration) associated with the secretion of enzymes to degrade skin components (10–12) (**Table 1**). Although there is a common understanding that dermatophyte keratinases are of major relevance for pathogenicity, the entire process of host adaptation during infection seems to be quite complex (15).

**Table 1:** Virulence factors of dermatophytes.

Virulence factor	Description and function	References
Subtilisin-like proteases (Sub)	Endoprotease activity in keratin digestion. Reported as allergens and involved in immune response induction.	Woodfolk et al. (13). Gao and Takashima (14). Burmester et al. (15). Eymann et al. (16). Karami Robati et al. (17). Mehul et al. (18). Reviewed in Mercer and Stewart (9).
Fungalysin-like Metalloproteases (Mep)	Endoprotease activity in keratin digestion.	Burmester et al. (15). Eymann et al. (16).
Leucinaminopeptidases (Lap)	Exoprotease activity in keratin digestion.	Burmester et al. (15). Eymann et al. (16).
Dipeptidyl peptidases (Dpp)	Exoprotease activity in keratin digestion.	Burmester et al. (15). Eymann et al. (16).
Secondary metabolite production-associated enzymes	Polyketide synthase and non-ribosomal peptide synthetase.	Burmester et al. (15). Martínez et al. (19).
Cysteine dioxygenases	Sulfitolysis of keratin. Involved in triggering humoral immune response during infection.	Grumbt et al. (20). Eymann et al. (16). Reviewed in Mercer and Stewart (9).
Hydrophobins	Hydrophobin rodlet layer on conidial surface. Related to avoiding immune recognition by neutrophils.	Heddergott et al. (21). Eymann et al. (16).
Extracellular vesicles	Unknown cargo, probably virulence factors. Related with modulation of the host response.	Bitencourt et al. (22)
LysM proteins	Protein domains related to binding to skin glycoproteins. Possibly involved in immune evasion.	Martinez et al. (19). Kar et al. (23)
Heat shock proteins	Hsp 30, Hsp60, Hsp70. Associated with adaptation to human temperature, keratin digestion.	Reviewed in Martinez-Rossi et al. (24).
Other hydrolases and cell wall remodeling-associated enzymes.	Lipases, glucanases, chitinases, betaglusidases, mannosyl transferases. Many involved in triggering humoral immune response during infection.	Burmester et al. (15). Martínez et al. (19). Eymann et al. (16). Martins et al. (25).

The colonization by anthropophilic species in humans or zoophilic species in animals usually causes asymptomatic infections or, eventually, chronic infections with minimal inflammation response suggesting a specific adaptation to the host probably in favor of survival and transmission. On the contrary, human infections with geophilic or zoophilic dermatophytes occur with inflammatory-associated symptoms and, generally, self-resolve (**Figure 1**). Therefore, depending on the host and the infective species, dermatophytes might differentially express virulence factors and activate, or suppress, particular immune receptors and signaling pathways that eventually would determine their own persistence or elimination. Comparative genome studies in various dermatophyte species have revealed that there are few differences in gene regulation and post-transcriptional mechanisms among them, but whether those differences might be responsible for the host-specific adaptation remains largely unknown (26).

Several dermatophyte enzymes and proteins participate in the keratin degradation and keratinolytic proteases activity culminates in the onset and maintenance of the infection process (9, 26). However, the virulence factors that are particularly related to pathogenicity degree and host adaptation have not been precisely identified yet. In the process of keratin degradation by dermatophytes, Graser et al. (12) have described three consecutive steps: first, the sulfitolysis stage [e.g., mediated by cysteine dioxygenases (20)], that liberates sulfites that reduce cysteine disulfuric bridges of the compact keratin in the stratum corneum should be in cursive to produce polypeptidic soluble chains that can be sliced by endoproteases. Second, endoproteases activity (subtilisins, deuterolysins, and fungalysins) liberates long peptides that are substrate to exoproteases (nonspecific amino- or carboxypeptidase, and prolyl peptidases) which, finally, transform long peptides into amino acids and short peptides that can be effectively assimilated by hyphae. Supporting this, Burmester et al. (15) analyzed the secretome of *Arthroderma benhamiae* after *in vitro* growth on keratin and revealed that about 75% of secreted proteins were proteases (subtilisin-like serine proteases: Sub3, Sub4, and Sub7; fungalysine-type metalloproteases: Mep1, Mep3, and Mep4; leucine aminopeptidases: Lap1 and Lap2; dipeptidyl-peptidases: DppIV and DppV) with the remaining formed by hydrolases and proteins involved in carbohydrate metabolism. Consistently, subtilisin proteases genes expression was detected on 93% of dermatophytes isolated from human patients (17) and, in particular, subtilisin 6 (Sub6) has been reported as the main protease secreted by *Trichophyton mentagrophytes* during guinea pig infection and human onychomycosis (14, 18). Also, Sub6 was detected in clinical samples infected with *Trichophyton rubrum* and it was one of the main allergens that produce an IgE-mediated response in susceptible hosts (5, 13). In this regard, exoproteome analysis of three *Trichophyton* species more frequently isolated from patients (*T. rubrum, T. interdigitale and A. benhamiae*) showed that, at least, 31 proteases (peptidases, oxide-reductases and beta-glucosidases) were recognized by antibodies in patients’ sera, indicating that these proteins are antigens involved in triggering humoral immune response during infection (14, 16) (**Table 1**).

The ability of fungal-derived proteases to interfere with the host immune response has been demonstrated for other human pathogens. For instance, a metalloprotease released from *A. fumigatus* conidia facilitates early fungal immune evasion by cleaving complement proteins in the human host (27). Moreover, *Candida albicans* secretes aspartic proteases, that cleave pro-interleukin (IL)-1β to its biologically active form IL-1β (27) and promote inflammation, as well as candidalysin, a peptide toxin that damages epithelial membranes and triggers a danger response signaling pathway (28). Whether similar phenomena are also mediated by dermatophytes proteases is currently unknown.

On the other hand, the experimental evidence also suggests that dermatophyte pathogenesis involves mechanisms beyond the fungal machinery used for keratin degradation, including virulence factors like cell wall components and secreted products (**Table 1**). In this regard, the transcriptome profile analysis of *A. benhamiae* after *in vitro* interaction with human keratinocytes revealed that not only proteases were found to be differentially regulated but also genes associated with the synthesis of secondary metabolism molecules (polyketide synthase and non-ribosomal peptide synthetase), lipases and hydrophobin (hypA) (15). In a similar way to RodA hydrophobin of *Aspergillus fumigatus* (29), the hypA protein of *A. benhamiae* forms a hydrophobic rodlet layer that enables conidia to avoid recognition by immune cells at an early stage of infection (21). Furthermore, other genes associated to the host-fungi interaction, mainly through genomic and/or transcriptomic analysis, were proposed: genes related to heat shock proteins (24), other enzymes that participate in keratin degradation (hydrolases, glucanases, chitinases, mannosyl transferases) (25), ergosterol metabolism and reproduction, and LysM domain proteins (19, 23). Strikingly, various of these virulence factors might be transported to the extracellular space by extracellular vesicles (EVs), as it was demonstrated that *T. interdigitale* produces these structures *in vitro* (22).

Therefore, the dermatophyte molecules that drive skin invasion, trigger inflammation, or facilitate evasion during the infection process still remain poorly understood. Further comprehensive research on dermatophyte virulence factors is necessary to identify the main microbial mechanisms that mediate difficult-to-treat chronic infections or overly inflammatory responses.

## Dermatophyte Recognition and Activation of the Innate Immune System

Immune and non-immune cells sense fungi through their cell wall components (i.e. polysaccharides, glycoproteins), secreted extracellular molecules (i.e. peptide toxins) or intracellular content (i.e. DNA) through different pattern recognition receptors (PRR) which, upon ligation, transduce intracellular signals that promote fungal phagocytosis, respiratory burst, cytokines and chemokines release, phagocyte lysis, among others, and thereby shape immune responses (30, 31). PRR can be classified based on their structure and function: C-type Lectin Receptors (CLR), Toll-like Receptors (TLR), Nucleotide-binding and Oligomerization Domain (NOD)-like receptors (NLR), and Retinoic acid Inducible Gene (RIG)-like receptors (RLR). Until now, signaling pathways mediated by CLR, TLR and NLR have been described in the interaction with dermatophytes and are key modulators of host antifungal immunity.

### C-Type Lectin Receptors

C-type Lectin Receptors (CLR) comprise a superfamily of soluble and membrane-bound proteins characterized by the presence of, at least, one C-type lectin domain (CTLD), some of which act as a carbohydrate recognition domain (CRD). The fungal cell wall contains numerous structures like glycans, glycolipids and glycoproteins that are recognized by several CLR (30) including Dectin-1 (CLEC7a), Dectin-2 (CLEC6a), Dectin-3 (CLEC4d), MINCLE (CLEC4e), Mannose Receptor (CD206), DC-SIGN (CD209), etc (32, 33). Dectin-1 recognizes β-glucans in the cell wall of diverse pathogenic fungi and is the best-characterized receptor involved in antifungal immunity. Dectin-1 signaling involves an immunoreceptor tyrosine-based activation motif (ITAM)-containing cytoplasmic domain that is phosphorylated by a Src family kinase that allows Syk kinase recruitment. Dectin-2 recognizes α-mannans and transduces its signal through association with the ITAM-containing Fc receptor gamma (FcRγ) chain (34). In most CLR signaling, the Syk pathway activates a molecular scaffold composed by CARD9, Bcl10 and MALT1, which culminates in the recruitment of several transcription factors including NF-κB and MAP kinases. Additional intracellular pathways are also induced and include the Raf-1 kinase pathway and the canonical (NLRP3/caspase-1) and non-canonical (MALT1/caspase-8) inflammasome activation pathways (30, 33).

By using CLR soluble fusion proteins, Sato et al. (35) demonstrated that Dectin-1 and Dectin-2 bind to *Microsporum audouinii* and *Trichophyton rubrum* while Dectin-2 particularly recognizes high mannose structures or oligo-manosid residues in the hyphae cell wall. This seminal work described that Dectin-2 couples FcRγ chain in RAW cells (human macrophage cell line) to trigger innate immunity after ligation by fungal hyphae. The activation of myeloid cells *via* CLR was further observed *in vivo* after *T. rubrum* intraperitoneal infection of C57BL/6 mice (36, 37). In this setting, WT mice controlled systemic infection at 14 days showing a significant decrease in spleen fungal burden whereas mice deficient in Dectin-1 or Dectin-2 (Dectin-1 or Dectin-2 knock-out) and double knock-out (KO) counterparts were unable to do the same. In addition, other studies showed that soluble α-mannans (33) and mannose receptor (CD206) blocking antibody (38) inhibited *T. rubrum* conidia engulfment by macrophages *in vitro*.

In a human disease context, Ferwerda et al. (39) first reported a family with defective surface expression of mutated Dectin-1 and susceptibility to chronic vulvovaginal candidiasis and persistent onychomycosis by *T. rubrum*. In these patients, peripheral blood mononuclear cells poorly expressed Dectin-1 and were deficient in producing tumor necrosis factor (TNF), IL-6, and IL-17, after stimulation with β-glucans or *Candida albicans*. According to this, individuals with inherited deficiencies in CARD9, the CLR downstream adaptor molecule, are also susceptible to severe deep dermatophytosis (40).

C-type lectin receptors expression, and its downstream Syk-CARD9 signaling, is primarily restricted to myeloid cells like monocytes, macrophages, neutrophils and dendritic cells (30), but it has also been described in epithelial cells (41) and keratinocytes (42). Nevertheless, few studies have investigated the role of CLR-expressing cell populations in the skin during dermatophytosis. In a mouse model of dermatophyte antigen-induced contact hypersensitivity (CHS), the percutaneous application of trichophytin (a soluble antigen from *T. mentagrophytes*) upregulated Dectin-1 mRNA (messenger RNA) expression in skin tissue and Dectin-1-expressing cells were involved in trichophytin-induced CHS (39, 40). Furthermore, Dectin-1 mRNA expression was up-regulated in HaCaT cells (a human keratinocyte cell line) co-cultured with supernatant from *T. rubrum* culture (43). In contrast, Brasch et al. (44) studied Dectin-2 expression by immunohistochemistry in patients with dermatophytosis (*tinea corporis*) and did not observe significant differences in Dectin-2 expression in the skin tissue between patients and healthy control individuals.

Therefore, the current knowledge strongly suggests that Dectin-1, Dectin-2 and Mannose Receptor expressed on myeloid cells play a role in triggering the anti-dermatophytic defense. Nonetheless, the function of particular CLR on skin cellular subsets driving antifungal response has not been defined in the context of cutaneous infection yet.

### NLRP3 Inflammasome and IL-1β Production in Fungal Infections

IL-1β is a potent inflammatory cytokine mainly produced by macrophages and neutrophils that promotes cytokine production, phagocytosis, oxidative burst and neutrophil degranulation. IL-1β is a cytokine produced as an inactive intracellular precursor triggered by PPR recognition of microbial pathogen-associated (PAMPs) or damage-associated (DAMPs) molecular patterns and later activated into the biologically active form by caspase-dependent cleavage after inflammasome assembly (45). The core of the majority of the inflammasomes is the NOD-like receptor (NLR) and the NLR family pyrin domain-containing 3 (NLRP3) is the most studied in fungal infections (46). IL-1β production *via* the inflammasome canonically requires two signals: the first is NF-κB-dependent activation provided by microbial binding to CLR or TLR that induces pro-IL-1β synthesis and NLRP3 transduction. The second signal is given by K^+^ efflux, extracellular ATP (adenosine triphosphate), reactive oxygen species (ROS), fungal toxins, or particulate matter, etc (31). Consequently, the second signal promotes NLRP3 activation by triggering the assembly of a multiprotein complex composed of NLRP3, the adapter protein ASC and the caspase-1 pro-form. This complex serves as a platform for pro-caspase-1 activation and, thereby, facilitates proteolytic pro-IL-1β processing to mature IL-1β (31, 46).

*Microsporum canis* and *Trichophyton schoenleinii* hyphae have been demonstrated to induce IL-1β production by THP-1 cells (a human monocytic cell line) and murine dendritic cells in a NLRP3 dependent-manner (47, 48). In addition, Dectin-1-Syk-CARD9 signaling was critical for pro-IL-1β transcription induced by *M. canis*, suggesting that dermatophyte glycan recognition by CLR provides the first signal for NLRP3 and IL-1β synthesis. Importantly, *M. canis* also triggered IL-1β production *in vivo*, after intraperitoneal infection of WT mice, but IL-1β release was completely abolished in NLRP3- or ASC-deficient mice (47). In line with these studies, *T. rubrum* conidia phagocytosis also induced IL-1β *via* Dectin-1 and Dectin-2, in a NLRP3-ASC-caspase-1 dependent-manner (36) and IL-1 signaling in macrophages restricted *T. rubrum* conidia germination and hyphae growth (37). The second signal proposed for NLRP3 activation and IL-1β release by *M. canis* or *T. schoenleinii* was found to be dependent on cathepsin B activity, K^+^ efflux and ROS production. Nevertheless, the dermatophyte-derived molecules that trigger this second signal remain unknown and, the only evidence, so far, is that it can be mediated by heat-sensitive molecules (47, 48). In this sense, dermatophyte heat-sensitive proteases might play a similar role as the secreted aspartic proteases (SAPs) from *C. albicans* which activate NLRP3 after its internalization *via* a clathrin-dependent mechanism with intracellular induction of K^+^ efflux and ROS production (49).

Taken together, dermatophyte activation of NLRP3 inflammasome *via* CLR on myeloid cells represents a key event for triggering innate immunity. Experimental skin infection with *Arthroderma benhamiae, A. vanbreuseghemii* (50) or *M. canis* (51) elicited IL-1β production by epidermal cells, however, the role of inflammasome-dependent antifungal immunity during skin dermatophyte infection is currently unknown.

### Toll-Like Receptors

Toll-like receptors (TLR) are membrane glycoproteins that were first described by their ability to control fungal infections in *Drosophila* and later were found to mediate mammalian host response against microbial pathogens. Upon ligand binding, TLR intracellular signaling is mediated by myeloid differentiation primary response 88 (MyD88) and TIR domain-containing adapter-inducer interferon-β (TRIF) to trigger the inflammatory response. Fungal ligands that bind to TLR are not completely defined, however, experimental evidence suggests that TLR cross-signal together with CLR and modulate the antifungal defense (30). In this regard, it has been demonstrated that TLR-2 increases its ligand-binding spectrum by heterodimerization with Dectin-1 for β-glucan recognition (52).

The studies concerning the role of TLR in the anti-dermatophyte immune response have produced results showing pro-inflammatory as well as anti-inflammatory effects that need further elucidation. Myeloid cells, keratinocytes and fibroblasts increase TLR-2 and TLR-4 mRNA expression upon interaction with dermatophytes (43, 53). *In vitro* studies with feline neutrophils showed an increase in TLR-2 and TLR-4 mRNA levels after stimulation with live and heat-killed *M. canis* arthroconidia (54). Recently, Celestrino et al. (55) reported that TLR-2 is important for *T. rubrum* conidia phagocytosis and pro-inflammatory cytokines production by human monocytes. Additionally, considering that TLR-2 is not a phagocytic receptor, these authors suggested that TLR-2 enhances CLR-mediated phagocytic activity.

Interestingly, extracellular vesicles (EVs) produced by *Trichophyton interdigitale* have been demonstrated to induce proinflammatory mediators by bone marrow-derived macrophages and keratinocytes in a TLR-2-dependent manner (22). The EVs are spherical structures composed of a lipid-bilayer membrane produced by different microorganisms and play a role in the secretion of virulence factors. Therefore, this study showed that dermatophytes can modulate the host innate immune response by producing EVs loaded with still undefined dermatophyte virulence factors that interact with TLR.

On the other hand, published data using a deep dermatophytosis model in TLR-2 deficient mice subcutaneously infected with *T. mentagrophytes* demonstrated that fungal interaction with TLR-2 suppresses the inflammatory response of peritoneal macrophages and the production of IL-17, IL-10 and IFN-γ (interferon-gamma) by splenocytes (56). Coincidentally, we observed a lower fungal burden in the skin of TLR-2 deficient mice compared to WT using an epicutaneous model of *M. canis* infection (Beccacece I., unpublished data). According to this, Netea et al. (57) observed that TLR-2 deficient mice were more resistant to disseminated candidiasis than WT, as there was increased chemotaxis and enhanced candidacidal capacity of TLR-2^-/-^ macrophages due to a more robust Dectin-1-mediated immune response in the absence of TLR-2. Furthermore, TLR-2 expression apparently suppresses Dectin-1-dependent production of CXCL8 (IL-8) against fungal β-glucans (52, 58). CXCL8 is a member of the CXCL chemokine family primarily involved in neutrophil recruitment and activation in response to tissue damage or infection (59) and can be directly induced in human keratinocytes by dermatophytes (53, 60).

In patients with dermatophytosis, TLR-2 and TLR-4 immunohistochemical staining was observed in lower epidermis from infected skin (44). Accordingly, a recent study demonstrated that *T. benhamiae* induced *in vitro* TLR-2 expression in human keratinocytes and dermal fibroblasts (53). Thus, so far, there is no clear evidence on the fungal molecules and mechanisms involved in TLR-dermatophyte interactions but, as many intracellular signaling molecules are shared between PRR pathways, it seems that a functionally important cross-talk with CLR might be crucial for the antifungal response outcome. The *in vivo* studies suggest a role of TLR-2 in downmodulating inflammation during dermatophytosis.

## Dermatophyte Interaction With Skin Cells in the Context of Infection

**Figure 2:**
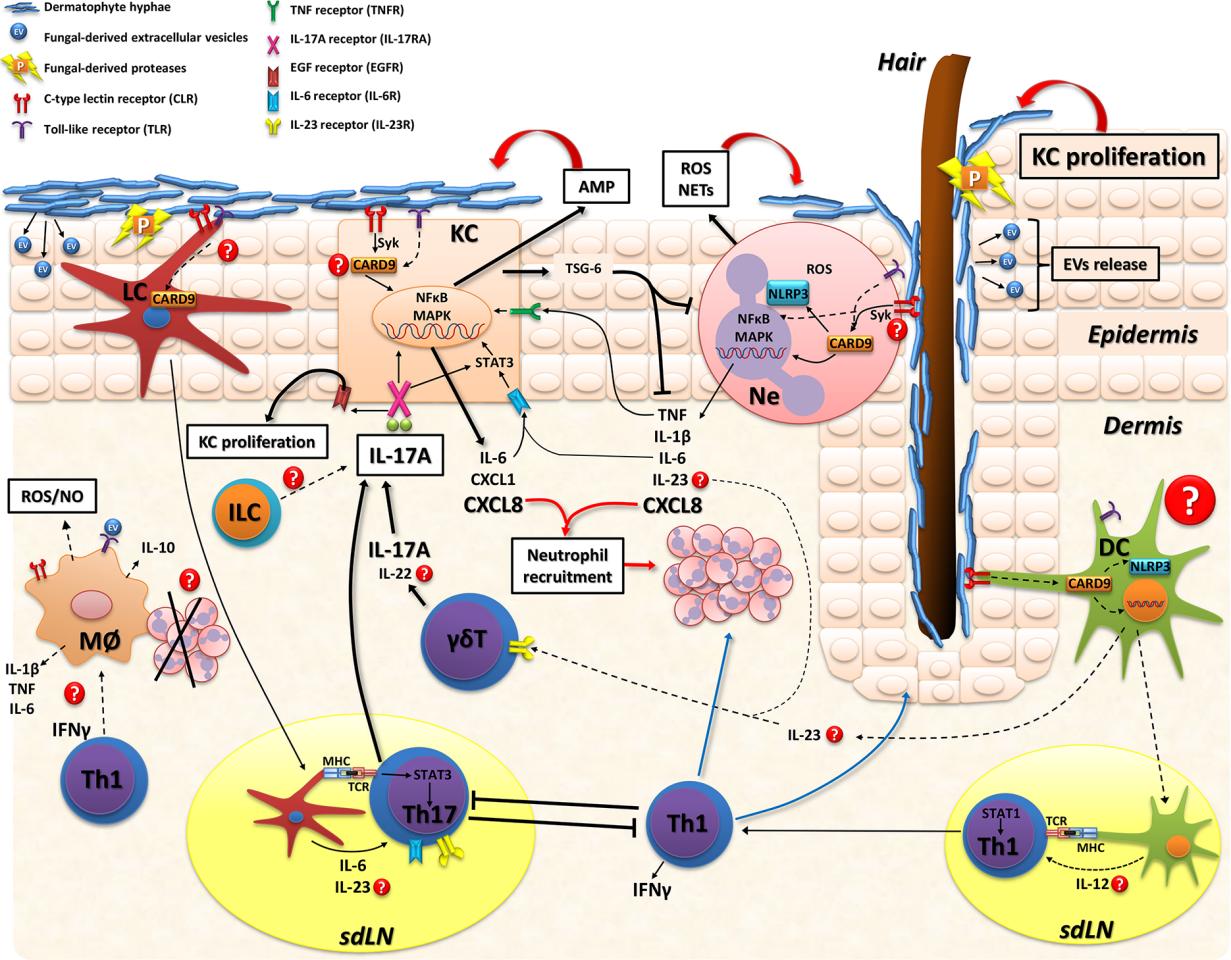
Model of skin immune response in dermatophytosis. Dermatophyte fungi invade the *stratum corneum* and release proteases (P) that degrade keratin for fungal growth and facilitate tissue invasion. Extracellular vesicles (EV) loaded with fungal virulence factors might be also released during infection. Host recognition of dermatophytes is mainly through CLR and TLR on myeloid cells and keratinocytes. The adapter protein CARD9 is a key molecule in fungal sensing that signals downstream various CLR and mediates cross-signaling of other innate receptors (TLR and NLR). Keratinocytes (KC) sense fungal hyphae and consequently release: 1) antimicrobial peptides (AMP; including cathelicidin and β-defensin) that are effector molecules promoting fungal clearance, 2) proinflammatory mediators (IL-6, CXCL8, TNF) that further stimulate inflammation and neutrophil recruitment or 3) immunosuppressive proteins, such as TSG-6, particularly in human infections with anthropophilic dermatophytes. Also, IL-6, IL-17, and IL-22 further stimulate KC activation. Neutrophils recognize fungi and trigger the intracellular activation of MAPK and NFκB pathways leading to proinflammatory cytokines/chemokines release that also enhance KC activation, recruit more inflammatory leukocytes and, probably, promote IL-17 producing-lymphocytes through IL-23 release. Neutrophils also secrete reactive oxygen species (ROS) and neutrophil extracellular traps (NETs) that kill dermatophytes. The role of macrophages has not been directly evaluated during skin infections but these cells might also kill dermatophytes by IFN-γ-induced ROS and nitric oxide (NO) production or even resolve inflammation through phagocytosis of apoptotic neutrophils with production of anti-inflammatory cytokines (e.g.: IL-10). As for adaptive immunity, Langerhans cells (LC) are located in the epidermis and sense dermatophytes, migrate to skin draining lymph nodes and promote Th17 differentiation (ref. 51). Whereas in the dermis, different subsets of dendritic cells (DC) are probably involved in sensing fungal molecules and producing cytokines that drive IL-17- or IFN-γ- mediated immunity. IL-17A produced by adaptive Th17 cells and innate lymphocytes (γδT or ILC) boosts KC activation/proliferation and inhibits superficial fungal growth. Upon binding to IL-17RA/IL-17RC in KC, IL-17A activates Act1-TRAF6-NFkB/MAPK or STAT-3 intracellular pathways and induce cytokines/chemokines and AMP production. Furthermore, the IL-17/IL-17RA pathway transactivates epidermal growth factor receptor (EGFR) which promotes KC proliferation. During mild superficial experimental infection with *M. canis*, type 17 immunity restricts both fungal growth and an exacerbated type 1 (IFN-γ mediated) inflammation (ref. 51). Conversely, IFN-γ mediated-response suppresses cytokines related to the IL-17 pathway leading to an increased fungal burden. During *T. benhamiae* experimental infection both Th1 and Th17 phenotypes are induced and control the cutaneous mycoses (ref. 120). Dotted lines and question marks refer to mechanisms not directly demonstrated in the context of skin dermatophytosis. CARD9 signaling might be involved in various skin cells populations of the antifungal defense. KC, keratinocyte; LC, Langerhans cell; DC, dendritic cell; Ne, neutrophil; ILC, innate lymphoid cell; Mø, macrophages; sdLN, skin draining lymph node; CLR, C-type lectin receptor; TLR, Toll-like receptor; NLR, nucleotide-binding oligomerization domain (NOD)-like receptor; IL-17RA/IL-17RC, interleukin 17 receptor A/C; TCR, T cell receptor; MHC, major histocompatibility complex; AMP, antimicrobial peptides; ROS, reactive oxygen species; NO, nitric oxide; NETs, neutrophil extracellular traps; P, proteases; EV, extracellular vesicles.

The *stratum corneum*, the outer layer of the skin, is composed of dead keratinocytes, keratin and hydrophobic lipids along with antimicrobial peptides (AMP), and function as a barrier against the environment and potential pathogens. In the epidermis, keratinocytes are essential to initiate the cutaneous immune response since they express various innate receptors (TLR, CLR, NLR, etc.) that detect pathogens and induce cytokine, chemokine, and antimicrobial peptide synthesis to locally modulate the recruitment and function of inducers and effectors cells from immunity. Furthermore, keratinocytes express cytokine receptors (such as IL-17R, IL-22R and TNFR) that potentiate this response. In between these cells, there are immune cell subsets strategically located to tissue immunosurveillance like Langerhans cells (LC) and resident memory CD8^+^ T cells; while in the dermis, there is a great variety of cell populations: different subsets of dermal dendritic cells (DC), macrophages, mast cells, innate lymphoid cells (ILC), γδ T cells and memory-resident and regulatory T cells (CD4^+^ and CD8^+^). Furthermore, there are nervous terminals that innervate the skin and lymphoid vessels from where immune cells migrate to lymphoid organs (61) (**Figure 2**).

Dermatophyte invasion is mostly restricted to keratinized tissues such as skin, hair, and nails but with the potential to cause extensive chronic superficial infections or even invasive systemic disease in immunocompromised patients. Widespread chronic or extracutaneous invasive infections have been largely related to patients with keratinization disorders or cell-mediated immunity deficiencies such as those observed in HIV, bone marrow and solid organ transplant recipients, or corticosteroid therapy (3, 5). In the last decade, inherited mutations in key signaling innate immunity pathways have been related to dermatophytosis susceptibility as well (40, 62, 63). Considering that our immune system exhibits tissue-specific restrictions (64) and the majority of results were obtained using myeloid cell populations, we scarcely understand the cellular and molecular mechanisms involved in the skin immune response to dermatophytes (**Figure 2**).

### Keratinocytes: Active Initiators of Cutaneous Immunity Response

The first epidermal cells encountered during the dermatophyte infection process are keratinocytes (60, 65, 66). Upon exposure to dermatophytes, keratinocytes release pro-inflammatory mediators such as IL-6, CXCL8, TNF, and AMP including cathelicidin and β-defensins (53, 66, 67). Tani et al. (60) demonstrated that CXCL8 was detected in supernatants of 24 h co-culture of normal human epidermal keratinocytes (NHEKs) with microconidia from *T. mentagrophytes*, *T. rubrum*, or *T. tonsurans*. Cytokine production was strongly enhanced with the zoophilic *T. mentagrophytes*, whereas lower levels were seen with the other anthropophilic species. Moreover, only *T. mentagrophyte*s induced TNF and granulocyte-macrophage colony-stimulating factor (GM-CSF) production by NHEKs. These results support clinical evidence from human dermatophytosis showing that zoophilic dermatophytes induce more severe inflammatory responses than anthropophilic species. As described above for NLRP3 activation in myeloid cells, heat-killed fungi were unable to induce cytokine production by NHEKs, suggesting that metabolic activity or products secreted by dermatophytes are necessary to trigger the inflammatory response. Similar findings were recently reported by Faway et al. (66) using reconstructed human epidermis (RHE), an *in vitro* three-dimensional model of human skin. After infection with *T. rubrum* arthroconidia, hyphae invaded the *stratum corneum* and located in the whole intercellular space altering the epidermal barrier integrity and activating keratinocyte response with mRNA expression and release of CXCL8, TNF-stimulated gene 6 protein (TSG-6), AMP (human β-defensin-2 and -3 and S100A7) and, in a lesser extent, IL-1α, IL-1β, TNF, thymic stromal lymphopoietin (TSLP), and granulocyte colony-stimulating factor (G-CSF). In line with these data, Hesse-Macabata et al. (53) have recently described that *T. benhamiae* promotes expression and secretion of pro-inflammatory cytokines/chemokines as well as expression of various AMP, TLR-2 and proliferation marker Ki67 after infection of human keratinocytes and dermal fibroblast.

Taken together, these *in vitro* models demonstrate that, in the absence of immune cells, keratinocytes not only form a physical barrier against dermatophyte invasion but also actively confer an early antifungal defense by triggering a skin-specific immune response (**Figure 2**). It is worthy to notice that experiments using reconstructed human epidermis (RHE) also produced high levels of the immunosuppressive protein TSG-6 after *T. rubrum* infection (66), indicating that keratinocytes could also provide mechanisms involved in inflammation control and tissue repair (68).

### Neutrophils

During dermatophytosis, neutrophils are the first leukocytes recruited to the site of infection (51, 69) and are thought to be responsible for fungal elimination from the skin (21, 70, 71) (**Figures 1** and **2**). In human infection, as well as in experimental dermatophytosis models, neutrophils form epidermal microabscesses surrounding the hyphae in the *stratum corneum* (50, 51, 72, 73) (**Figure 1**). According to this, CD11b^+^ Ly6G^+^ neutrophils can be detected by flow cytometry in epidermal cell suspensions as early as 2 days after *M. canis* epicutaneous infection in mice (51). Early studies performed by Calderon and Hay (70) demonstrated that human peripheral blood neutrophils exhibited potent cytotoxic activity against *T. quinckeanum* and *T. rubrum* hyphae. Moreover, dermatophyte-stimulated human neutrophils were able to phagocyte conidia, produce CXCL8, IL-1β, IL-6, IL-8, and TNF, and stimulate extracellular traps (NETs) formation (21, 74).

Despite all the experimental evidence suggesting a main role of neutrophils as the first ‘defenders’ against dermatophytes, neutropenic patients are not frequently susceptible to extracutaneous invasive infection as they are to other fungi (including normally non-pathogenic species) (75). Instead, neutropenic patients frequently present widespread superficial infections and dermal granulomas resistant to antifungal treatment (Majocchi’s granulomas) (76, 77). In agreement with this, De Sousa et al. (74) studied a cohort of patients with chronic widespread dermatophytosis without noticeable predisposing conditions or signs of immunodeficiency and found that their blood-derived neutrophils and macrophages presented impaired *in vitro* killing of *T. rubrum*. Additionally, neutrophils from patients displayed defects in hydrogen peroxide, nitric oxide and cytokine production compared to neutrophils from patients with typical dermatophytosis (*tinea pedis*) or healthy donors. However, this study did not further demonstrate the cause of antifungal defects showed by the phagocytic cells.

Furthermore, impairment in neutrophil mobilization to the site of infection or fungal killing mechanisms could be associated with inherited CARD9 deficiency and dermatophytic disease in humans (78, 79). Nevertheless, to which extent neutrophils control cutaneous defenses against dermatophytes and restrain extracutaneous fungal invasion remains not fully understood.

### Antimicrobial Peptides

Antimicrobial peptides and proteins (AMP) are innate immune effector molecules that not only have microbicidal activity but also function as chemoattractants and proteinase inhibitors, have proangiogenic activity, promote wound repair, and can modulate adaptive immunity. The importance of AMP as antimicrobial effector molecules relies on fast killing mechanisms like forming pores in the microbial cell wall or nutrient depletion by extracellular Zn^2+^ and Mn^2+^ chelation (80, 81).

In the skin, there are more than 20 AMP including human β-defensins (hBDs), cathelicidins (LL-37), S100 proteins, RNase 7 and lactoferrin, among others (82). Keratinocytes constitutively express hBD1 whereas hBD2, hBD3, and hBD4 require TLR signaling or induction through TNF, IL-1β, or other cytokines (83). Additionally, other signaling pathways also regulate AMP production by keratinocytes, such as insulin-like growth factor 1 (IGF-1), vitamin D signaling and epidermal growth factor receptor (EGFR) ligands (83). In this regard, EGFR regulates transcription of downstream AMP genes (hBD-2 and -3, RNase 7, S100A7, elafin) and several cytokines and chemokines (CXCL8, IL-6, IL-20, IL-24, CXCL1) (80, 82). Keratinocyte stimulation of G-coupled receptors and certain cytokine receptors (such as IL-17RA/IL-17RC) results in matrix metalloproteinase (MMP)-mediated shedding of EGFR ligands (amphiregulin and heparin-binding epidermal growth factor-like growth factor, HB-EGF, and transforming growth factor-alpha, TGF-α) leading to transactivation of EGFR and further AMP expression in the skin (**Figure 2**). Strikingly, EGFR blocking in keratinocytes significantly decreased AMP expression in response to *T. rubrum* which could explain the increased dermatophyte infection rate observed in patients receiving therapy with anti-EGFR (81).

Lopez-Garcia et al. (84) firstly reported that cathelicidins may play a role in the skin defense against dermatophytes. They detected increased cathelicidin protein expression in the skin from patients with *tinea pedis* and *in vitro* inhibition of *Trichophyton* sp. growth by cathelicidin-derived synthetic peptides LL-37 and CRAMP. Similar data were later reported by Brasch et al. (44), demonstrating that AMP psoriasin (S100A7), hBD-2 and RNase 7 also inhibit dermatophyte growth *in vitro* (85). In agreement, Firat et al. (81) published that human foreskin-derived keratinocytes exposed to *T. rubrum* strongly boosted RNase 7 and hBD-3 expression and this phenomenon was synergistically increased in the presence of IFN-γ and IL-17A. Moreover, Sawada et al. (67) observed that expression of epidermal hBD-2 and LL-37 was significantly lower in Adult T-cell leukemia/lymphoma (ATLL) patients with dermatophytosis than in infected non-ATLL patients, and this correlated with a significantly decreased frequency of peripheral T helper 17 (Th17) lymphocytes and lower IL-17 levels in serum. The ATLL immune condition revealed that Th17 cells are deeply involved in keratinocyte production of antimicrobial peptides against dermatophytes.

## Type 17 (IL-17-Mediated) Immunity in Dermatophytosis

Type 17 immunity have a critical role in innate and adaptive immunity at barrier tissues such as oral, intestinal and lung mucosa as well as the skin. IL-17 cytokines play a major role in maintaining local homeostasis with microbiota, protecting against infections and mediating severe inflammatory diseases such as inflammatory bowel disease or psoriasis (86–88). IL-17 family members comprise six related proteins: IL-17A, IL-17B, IL-17C, IL-17D, IL-17E (IL-25), IL-17F, and the heterodimer IL-17AF. Among them, IL-17A has been the most studied cytokine and, therefore, associated with human health and disease (86). IL-17A is produced by hematopoietic cells from both innate and adaptive immune system, including CD4^+^ T helper (Th17), CD8^+^ cytotoxic T (Tc17), γδT, natural killer (NK), group 3 innate lymphoid (ILC3), and ‘natural’ Th17 cells. Members of the IL-17 family act like “local cytokines” mainly on non-classical immune cells such as epithelial, endothelial, and fibroblastic cells. IL-17 signals through heterodimeric receptors composed of the subunit IL-17RA associated with either IL-17RC, IL-17RE, or IL-17RB, which are specifically stimulated by IL-17A and F, IL-17C, and IL-17E (IL-25), respectively (89). Upon signaling on keratinocytes, IL-17 stimulates the production of various cytokines (such as GM-CSF, TNF, IL-6), chemokines (CXCL1, CXCL8), and vascular endothelial growth factor (VEGF). IL-17 can also enhance the expression of AMP such as hBD-2 and LL-37 and promote keratinocyte proliferation (80). All these mechanisms may play a major role in clearing cutaneous fungal infection (90, 91) (**Figure 2**).

In the skin, fungal recognition by PRR on myeloid cells (DC, macrophages, neutrophils) and, probably, keratinocytes and fibroblasts trigger the production of cytokines like IL-23, IL-6, IL-1β, and IL-21 (51, 53, 90). Cytokine binding to its receptor on lymphocytes selectively triggers intracellular Signal Transducer and Activator of Transcription 3 (STAT3) phosphorylation, Retinoic-acid-receptor-related orphan nuclear receptor gamma (RORγt) transcription factor activation and, eventually, leads to the induction of Th17 lineage or the synthesis of type 17-cytokines (IL-17, GM-CSF, IL-22) (92). Noteworthily, IL-17A can also activate STAT3 in keratinocytes and amplify IL-6 production (80) (**Figure 2**). In line with this, patients with STAT3 mutations (Autosomal dominant hyperimmunoglobulin E syndrome, AD-HIES) have increased susceptibility to candidiasis and dermatophytosis due to a diminished Th17 response (62).

It is well known that mucocutaneous candidiasis (CMC), a chronic and recalcitrant clinical syndrome, is associated with several genetic diseases related to dysregulation or inhibition of the IL-23/IL-17 pathway (33, 93). In this sense, IL-17-producing memory CD4^+^ T cells in peripheral blood expand specifically upon *Candida albicans* stimuli (94) and skin resident Th17 lymphocytes mediate protective immunity to this yeast (95). However, much less is known about the skin T cell response during dermatophytosis. Deficiencies in type 17 immunity associated with susceptibility to widespread chronic dermatophytosis have been reported in patients with ATLL (67), autoimmune polyendocrinopathy-candidiasis-ectodermal dystrophy (APECED) (72), Dectin-1 mutations (39), loss-of-function mutations in STAT3 (62), autosomal gain-of-function mutations in STAT1 (96, 97), and anti-IL-17 antibody treatment (secukinumab) (73, 98). Furthermore, IL-17 impairment was also associated with inherited CARD9 deficiency and deep dermatophytosis (40, 99).

## Experimental Dermatophytosis as Models of Human Disease

In contrast to research on *Candida* sp. infection, the lack of murine experimental models for dermatophytosis that mimic natural human infection long-hindered the possibility of in-depth studies on immunological mechanisms involved in the susceptibility to these mycoses. In the 1980s, Hay et al. (100, 101) made important contributions to the understanding of the anti-dermatophytic immunity by developing a highly inflammatory dermatophytosis model, similar to favus in humans, after epicutaneous infection of BALB/c mice with the murine pathogen *T. quinckeanum*. They showed that mice self-controlled the infection with a peak of skin lesions at 7–10 days, characterized by epidermal proliferation (acanthosis), neutrophil recruitment (epidermal microabscesses) and increased antigen-specific lymphocyte proliferation (69). Furthermore, they found that the adoptive transfer of T lymphocytes conferred resistance to sub-lethally irradiated mice (101) and that immunological memory was evidenced in re-infected mice (secondary infection) as they quickly cleared fungi and showed an augmented lymphoproliferative response to fungal antigens (100).

After almost 30 years of the studies published by Hay and colleagues, in 2014, Cambier et al. (50) developed a similar model in C57BL/6 mice with the zoophilic species *Arthroderma benhamiae* and *A. vanbreuseghemii* (both from the *Trichophyton mentagrophytes* complex) that cause highly inflammatory dermatophytosis in humans (102, 103). In this regard, experimental models of human diseases developed in a C57BL/6 mice background are remarkably important for the study of immunological mechanisms due to the wide variety of genetically modified strains available (104). Mice epicutaneously infected with *Arthroderma* self-healed at approximately 30 days with a clinical course and features similar to the inflammatory dermatophytosis in humans (50). Interestingly, *Arthroderma*-infected skin showed mRNA overexpression of proinflammatory cytokines like transforming growth factor-beta (TGF-β), IL-1β, IL-6, and IL-22 at days 7 and 21 post-infection, suggesting a role of type 17-immunity in host defense. In this line, we later demonstrated that IL-17-mediated immunity is key for host protection in an experimental epicutaneous infection of C57BL/6 mice with *Microsporum canis* (51), a zoophilic dermatophyte that causes highly prevalent *tinea capitis* and *tinea corporis* in children (105–107) (**Figure 1**). Similarly to the *Arthroderma* model, *M. canis*-infected mice showed skin lesions only limited to the epidermis and hair follicles, with a maximal clinical score at 8 days post-infection and fungal clearance around day 28. However, in contrast to that observed in *Arthroderma*-infected mice, *M. canis* infection was characterized by mild cutaneous lesions resembling non-inflammatory human disease (**Figure 1**). Re-stimulation of skin draining lymph node cells with heat-killed *M. canis* showed that Th17 adaptive immunity predominates during infection (51). As demonstrated for *Candida* and *Malassezia* (108, 109), *M. canis* selectively triggers a type 17 immune response mainly by CD4^+^T and, to a lesser extent, by CD8^+^T lymphocytes in lymph nodes around 8 days post-infection. Furthermore, IL-17-deficient mice (IL-17RA KO or IL-17A/F KO) were extensively colonized with *M. canis* hyphae as demonstrated by a forty-fold increase in skin fungal burden compared to WT infected mice (**Figure 1**). IL-17-deficient mice showed less epidermal thickening (acanthosis) nevertheless, pro-inflammatory cytokine production was up-regulated after infection. Moreover, in the absence of a functional IL-17 pathway, *M. canis* did not invade dermis or deep tissues in mice (51) in coincidence with clinical evidence showing that humans with deficiencies in type 17 immunity or treated with anti-IL-17 antibody (secukinumab) are susceptible to widespread superficial infections rather than deep dermatophytosis (67, 73).

Strikingly, in our model, neutrophil function and mobilization to the site of infection were uncoupled from IL-17 signaling since IL-17-deficient mice had a significantly higher frequency of neutrophils in the skin (51) (**Figure 1**). Supporting these findings, neutrophilic microabscesses were observed next to hyphae in the epidermis of a patient with dermatophytosis after 4 weeks of treatment with anti- IL-17 antibody (73). Further research is needed to establish the *in vivo* role of neutrophils in dermatophytosis and the molecular pathways driving its mobilization to the skin. Considering that dermatophytes induce a robust production of chemokines and cytokines (CXCL8, CXCL1, IL-6) by keratinocytes (53, 60, 65, 110), these chemotactic factors are probably the main mediators of neutrophil recruitment, independently of IL-17 signaling.

Altogether, these experimental and clinical results reveal that type 17 immunity is important to boost keratinocyte proliferation and probably early production of AMP after dermatophyte skin invasion. Eventually, IL-17 deficient hosts are able to overcome infection but at the expense of an exacerbated inflammation and tissue damage (51, 111).

On the other hand, anthropophilic dermatophytes (e.g. *T. rubrum, T. interdigitale, T. tonsurans*) are evolutionarily adapted to human keratin and consequently, murine experimental models might have limitations in recapitulating the features of inflammation and specific immune response occurring in a natural setting. Nevertheless, Baltazar et al. (112) have reported some interesting data by developing a model of *T. rubrum* infection in C57BL/6 mice after epicutaneous and intradermal inoculation of conidia. Likewise to the *M. canis* model, *T. rubrum* infected mice showed a peak of fungal burden after 7 days of infection (112) that significantly decreased by day 14. Along with *T. rubrum* infection, myeloperoxidase (MPO) and N-acetylglucosamine (NAG) activity was detected in the skin, suggesting recruitment of neutrophils and macrophages to the site of infection. Interestingly, MPO activity decreased in parallel with fungal load (14 days post-infection), but NAG activity remained elevated, even after fungal clearance (112). The authors hypothesized that skin macrophages might be involved not only in fungal killing, but also in the resolution of inflammation. In this sense, phagocytosis of apoptotic neutrophils by macrophages is a key mechanism to down-modulate inflammation and return to tissue homeostasis during infectious diseases (113) (**Figure 2**).

## Type 1 (IFN-γ-Mediated) Immunity

The role of IFN-γ-mediated (type 1 or Th1) response in protective skin immunity against dermatophytes remains less clear than IL-17-driven immunity. In the *M. canis* model, dermatophyte infection in WT mice did not trigger the expansion of antigen-specific IFN-γ-producing T cells in skin draining lymph nodes (51). In contrast, IL-17-deficient mice experienced a shift to a type 1 response suggesting the establishment of IFN-γ-mediated compensatory mechanisms to restraint *M. canis* infection. Nonetheless, *in vivo* IFN-γ neutralization in IL-17RA KO mice (at days 3 and 6 post-infection) increased skin production of Th17-lineage cytokines (IL-22, IL-17, IL-1β, IL-6) and significantly inhibited fungal growth (51). These surprising data open the possibility that IFN-γ deregulation, in the absence of IL-17 signaling, might contribute to superficial *M. canis* overgrowth by inhibiting type 17-related responses. However, the mechanisms by which Th1 cytokines may interfere with cutaneous immunity against dermatophytes and counter-regulate the IL-17 pathway remain unknown. Therefore, in this model, type 17 immunity has a dual role during infection by inhibiting dermatophyte growth and controlling Th1-mediated inflammation (51, 111).

In line with the experimental data observed in the *M. canis* mouse model, patients with mutations that lead to the gain of function in the transcription factor STAT1 (STAT1 GOF), that promote type I and type II IFN genes transcription, have reduced type 17 immunity and are susceptible to chronic mucocutaneous candidiasis (CMC) and dermatophytosis (96, 97, 114–117). Consistent with this, increased STAT1 responses to Th1 cytokines (IFN-γ and IL-27) were shown to repress the differentiation of IL-17-producing T cells through mechanisms that are not yet completely understood (114, 118). In fact, increased STAT1 phosphorylation induced by IFN-γ can be reversed upon treatment with the JAK kinase inhibitor, ruxolitinib (113) and, interestingly, patients with STAT1 GOF condition treated with ruxolitinib have shown remission of mucocutaneous candidiasis (119). Altogether, the clinical data supports the experimental results from *M. canis* model (51), showing that dermatophyte susceptibility is mainly due to deficiencies in IL-17-driven immunity and that type 17 and type 1 immunity would eventually counter-regulate each other.

Nevertheless, Heinen et al. (120) observed that, after *Trichophyton benhamiae* epicutaneous infection in C57BL/6 mice, Th17 along with Th1 responses function in a complementary manner and, only when both IL-17 and IFN-γ pathways are deficient, mice suffer from superficial persistent infection. In contrast, Baltazar et al. (112) reported that IL-12p40 KO mice (lacking common β-subunit of IL-12 and IL-23 and thus, with impaired IL-17 and IFN-γ signaling) or IFN-γ KO mice were able to control *T. rubrum* infection after 14 days, but showed an increased fungal burden in the first week compared to infected WT mice. Eventually, as described for the *M. canis* model (51), deep dermatophytosis was not observed in the absence of IL-17 and IFN-γ in neither infection models (112, 120), suggesting that several immune pathways must be compromised to establish invasive dermatophytosis. The effector mechanisms of IFN-γ remains unclear but Verma and Gaffen (121) hypothesized that, as observed in *Candida* skin infection, IFN-γ may contribute to *T. benhamiae* destruction and expulsion by activating the fibrinolytic system in the epidermal abscess (122) or promoting M1 macrophages at a later infection stage (**Figure 2**). In line with this, peritoneal macrophages from IFN-γ KO mice showed decreased ROS production and were unable to efficiently phagocyte and kill *T. rubrum* conidia *in vitro* (112) indicating that macrophages might play a role in clearing infection.

The most noticeable difference between the *M. canis* (51) and the *T. benhamiae* (120) model is that mice infected with *T. benhamiae* had a more aggressive infection with significantly augmented fungal burden and skin inflammation, compared to *M. canis*-infected mice. Therefore, undefined specific virulence factors produced by these two pathogens may selectively activate different immune pathways in the skin. In addition, the route of infection in the *T. rubrum* model (intradermal conidia inoculation) might activate specific subsets of dermal dendritic cells that could eventually promote IFN-γ-mediated response (112) (**Figure 2**). This was observed in *Candida albicans* skin infection, where epicutaneous infection induces Th17 response through Langerhans cells (LC), while invasive *Candida* hyphae in dermis triggers Th1 immunity mediated by CD103^+^ dermal dendritic cells (108).

In conclusion, the experimental data confirm clinical evidence showing that type 17 immunity is crucial for preventing uncontrolled superficial dermatophyte growth and for restricting an exacerbated cutaneous inflammation. The function of type 1 immunity is less clear since it inhibits type 17-mediated protective mechanisms (in the absence of an optimal IL-17 signaling) as observed in the mild inflammatory *M. canis* model or, in contrast, it might contribute to clear the infection as observed in the *T. benhamiae* inflammatory model.

## Skin Antigen-Presenting Cells and Innate Versus Adaptive Response

In the steady-state skin, there is a coexistence of several antigen-presenting cells (APC) with the capacity to initiate and shape the immune response against pathogens. Langerhans cells are the most abundant population of skin APC and are constitutively localized in the epidermis. Other major populations of migratory skin DC are present within the dermis: CD103^+^ DC subset, CD11b^+^ DC (CD1c^+^ DC in humans) and double negative DC. The current consensus is that these defined DC subsets contribute differently to antimicrobial immunity depending on the pathogen, the site or the stage of infection (123). The paradigm of adaptive immunology poses that after pathogen invasion, skin DC capture antigens and migrate to lymph nodes to present them to T lymphocytes in an MHC-TCR (Major Histocompatibility Complex–T cell receptor) context, thereby expanding antigen-specific T helper or T regulatory clones which return to the skin to fight the infection and modulate inflammation, respectively. In *Candida albicans* or *Malassezia* epicutaneous infections, IL-6- or IL-23-producing LC migrate and induce an antigen-specific Th17 response by 7–10 days post-infection (90, 109). At the same time, cutaneous DC are also able to rapidly and locally activate resident skin cells to arm the innate immune response. Notably, *C. albicans* directly stimulates sensory neurons to produce a neuropeptide that, in turn, induces IL-23 production by dermal DC and promotes a rapid protective response by IL-17-producing γδT cells (124). Moreover, as early as 2 days during *Malassezia* infection, not only LC but also neutrophils produced IL-23 to stimulate IL-17A-production by γδT, ILC and αβT cells in the skin (109). Nonetheless, the precise mechanisms involved in the induction of protective immunity in dermatophytosis are far less clear. Type 17 immunity-instructing factors IL-1β, IL-6, TGF-β and IL-23 were produced by epidermal cells after *M. canis* and *T. benhamiae* dermatophytosis in mice (51, 120). In the *M. canis* model, langerin expressing-dendritic cells (LC and a minor population of dermal DC) contributed critically to the regulation of the *M. canis*-specific Th17 response in draining lymph nodes (51), which is reminiscent of Th17 induction in response to epicutaneous infection with *C. albicans* (125) and *Malassezia* (109). However, *M. canis* fungal burden was not affected by fungal-specific Th17 cell reduction after depletion of langerin-expressing DC (51), pointing towards alternative innate sources of IL-17 for controlling dermatophytosis (**Figure 2**). In this sense, Heinen et al. (120) observed that IL-17 mRNA expression was early induced (3 days post-infection) in the skin of *T. benhamiae*-infected mice and showed a remarkably strong contribution of innate immunity in clearing dermatophytes from the skin. They demonstrated that *Rag2^-/-^* mice (lacking T and B cells) presented a long-lasting infection but were ultimately able to clear *T. benhamiae* from the skin.

## Inherited CARD9 Deficiency and Deep Dermatophytosis

The clearest evidence of innate immunity involvement in restricting dermatophyte extracutaneous invasion was the discovery of loss-of-function mutations in the adapter protein CARD9 as the primary immunodeficiency underlying deep dermatophytosis (126). Deep dermatophytosis is a severe, recalcitrant and, sometimes, a life-threatening infection characterized by extensive invasion of the dermis (106) (**Figure 1**), hypodermis, and deeper tissues, by dermatophytes (6). As described above, CARD9 is a caspase recruitment domain-containing signaling protein crucial for CLR downstream signaling and gene activation by fungal glycans but it is also involved in cross-signaling with other innate receptors, such as TLR and NLR (107). Thus, CARD9 protein plays a critical role in innate immunity probably controlling various innate immune pathways (**Figure 2**).

In 2013, Lanternier et al. (40) first demonstrated that 17 non-consanguineous patients with deep dermatophytosis by *T. rubrum* or *T. violaceum*, had autosomal recessive CARD9 deficiency without other associated infectious conditions, except oral candidiasis in six of them. These authors described two CARD9 mutations: a homozygous premature stop codon mutation (Q289), identified in 15 patients from seven unrelated Algerian and Tunisian families, and a homozygous missense mutation (R101C) in two Moroccan siblings. The functional consequence of CARD9 mutations was a markedly low level of IL-6 production after stimulation of whole-blood leukocytes with heat-killed *C. albicans* or *S. cerevisiae*, but not with TLR agonists. Furthermore, peripheral Th17 cells from CARD9-deficient patients were significantly less frequent than healthy controls. CARD9 Q289 mutation was later described in Egyptian patients with widespread superficial *T. rubrum* infection of the skin and nails without significant visceral involvement (127). Recently, this mutation was also reported in an Algerian woman who suffered from cutaneous chronic dermatophytosis by *T. rubrum* from her childhood and developed an invasive brain infection in her adulthood (128).

The phenotypic variability of dermatophytic infection observed in patients with CARD9 deficiency ranges from extensive skin and nail lesions to potentially lethal lymph node and central nervous system infection. In 2015, Grumach et al. (129). reported a Brazilian patient with deep dermatophytosis by *T. mentagrophytes* harboring a novel CARD9 mutation, R101L. The patient displayed impaired fungal killing by neutrophils and low numbers of CD16^+^/CD56^+^ NK cells in peripheral blood. Similarly, Alves de Medeiros et al. (130) described a homozygous R70W CARD9 mutation in a Turkish family with resistant chronic cutaneous and deep dermatophytosis along with mucocutaneous and invasive candidiasis. In these patients, circulating IL-17 and IL-22 producing T cells were decreased as well as IL-6 and GM-CSF secretion by peripheral mononuclear cells upon stimulation with *Candida albicans*. Furthermore, high levels of serum IgE and eosinophilia were also a feature in all patients with CARD9 deficiency and invasive fungal invasion, but the link of these responses with the absence of CARD9 remains unexplained so far (40, 127, 130).

The cellular and molecular pathways related to CARD9 signaling in the skin have been scarcely investigated (131, 132) and it is currently unknown how CARD9 precisely drives cutaneous antifungal immunity (**Figure 2**). The accumulating evidence in humans has shown, so far, that susceptibility to fungal diseases in CARD9-deficient patients is related to the poor production of inflammatory cytokines by myeloid cells in response to fungal antigens along with an impairment in type 17-mediated immunity (126). Furthermore, CARD9 signaling was required in tissue-resident cells for an appropriate induction of CXC chemokines for neutrophil recruitment to the site of fungal infection (79). Noteworthily, Queiroz-Telles et al. (78) have recently reported a successful allogeneic hematopoietic stem cell transplantation (HSCT) in two patients with inherited CARD9 deficiency and deep dermatophytosis. More than 3 years after HSCT, both patients have achieved complete clinical remission and stopped antifungal therapy. This evidence points toward deep dermatophytosis pathogenesis in CARD9-deficiency settings might be largely due to the disruption of myeloid cell antifungal response.

## Conclusions and Future Directions

Over the last years, remarkable progress has been made for immunity to fungal pathogens while the physiopathogenesis of dermatophytosis remains poorly explored. So far, clinical and experimental evidence shows that type 17 immunity controls superficial infection, probably by promoting antimicrobial peptide production and keratinocyte proliferation, and independently of early recruited neutrophils with fungicidal function at the site of infection. Additionally, in the setting of mild inflammatory infections, IL-17-mediated response would be crucial to control cutaneous homeostasis preventing detrimental Th1 inflammation. The susceptibility to extracutaneous deep dermatophytosis would be related to deficiencies in various immunity pathways converging in CARD9 activation and probably restricted to the myeloid cell compartment.

In addition to causing symptomatic infections with different severity degrees (**Figure 1**), dermatophytes also colonize 30%–70% of the human population without causing clinical disease (1, 2, 133), thus these keratin parasites could be considered as a component of the human microbiota. Strikingly, the presence of dermatophyte in the skin, either in commensal or pathogenic relationship with susceptible hosts, has been related to asthma, allergy or eczematous skin (5, 13, 134). Therefore, the colonization of the skin by dermatophytes is an interesting concept to explore for its potential ability to induce migratory or tissue-resident immune cells that could participate in inflammatory pathological settings.

The increasing dermatophytosis incidence in certain geographic areas, the growing evidence of susceptible hosts with severe clinical presentations and the emerging antifungal resistance (7, 33, 135) highlight the need for a deeper understanding of the dermatophyte-host interaction. The next challenges are thoroughly defining which are the virulence factors responsible for dermatophyte pathogenesis and the mechanisms involved in limiting the infection, or, otherwise, those favoring chronicity or asymptomatic colonization.

## Author Contributions

The review results from the discussion and the consensus of all authors listed. The review was written by VLB and LSC. **Figure 2** was graphed by IB. All authors contributed to the article and approved the submitted version.

## Funding

The authors acknowledge financial support from SECyT-UNC, ANPCYT-PICT 2018-1349, and CONICET-PIP (GI11220150100260), Argentina. VLB is a postdoctoral fellow of CONICET, and LSC is a member of the scientific career of CONICET.

## Conflict of Interest

The authors declare that the research was conducted in the absence of any commercial or financial relationships that could be construed as a potential conflict of interest.
